# Microbiological and Physicochemical Variations during Spontaneous Fermentation of Plantain Must

**DOI:** 10.1155/2023/8611252

**Published:** 2023-09-13

**Authors:** Ollo Kambire, Konan Mathurin Yao, Karamoko Detto, Moustapha Kamate

**Affiliations:** Department of Biochemistry and Genetics, Peleforo Gon Coulibaly University, BP 1328 Korhogo, Côte d'Ivoire

## Abstract

Major losses are recorded every year in the plantain production sector in Côte d'Ivoire. These losses are mainly due to poor harvesting, transport, and storage conditions. Local processing of this food crop into other products could help limit postharvest losses. The aim of this study was to evaluate some microbiological and physicochemical parameters during the fermentation of plantain must for the production of an alcoholic beverage. Physicochemical parameters such as pH, titratable acidity, and reducing sugars were measured using a pH meter by titration and the Bernfeld method, respectively. Refractometric dry extract and alcohol content were measured using a refractometer. Loads of mesophilic aerobic germs, total coliforms, streptococci, and yeasts were determined by standard microbiological methods. Values for refractometric dry extract (10°B-4.5°B), reducing sugars (8.25-0 mg/mL), and pH (4.37-3.36) decrease during fermentation. The highest alcohol content (11%) is obtained after four days of fermentation of plantain must. In contrast to total coliforms (5.27-3.61 log_10_ cfu/mL), loads of mesophilic aerobic germs (4.84-9.8 log_10_ cfu/mL) increase during fermentation. Yeast and streptococci loads reach their peaks at 7.81 log_10_ cfu/mL and 8.15 log_10_ cfu/mL, respectively, after six (6) days of fermentation before dropping off. Plantain must could be used to produce distilled alcoholic beverages.

## 1. Introduction

At the dawn of independence, African countries were less endowed with human capital and technology than their northern counterparts. Soil fertility combined with good climatic conditions led them to turn naturally to the agricultural sector, which they saw as an engine of economic development. Côte d'Ivoire has not escaped this situation by installing an agrarian-type rentier regime [1]. Located in West Africa between 4°30 and 10°30 north latitude and between 2°30 and 8°30 west longitude, Côte d'Ivoire's economy is based on agriculture. This agriculture includes the production of plant resources as well as animal and fish resources. Two subsectors make up the plant resources sector: cash crops or industrial crops and food crops [2]. The main cash crops are coffee, cocoa, oil palm, and rubber. The food crop subsector employs 85% of the agricultural workforce, 90% of whom are women. Food crop production, estimated at 9,000,000 tonnes in 2006, occupies an area of 2,448,000 ha [2]. This subsector includes the following main crops: rice, yams, cassava, maize, vegetables, and plantains. Annual plantain production is estimated at 1.42 million tonnes, making Côte d'Ivoire the third largest plantain producer in West Africa, behind Nigeria and Ghana [3]. It has developed thanks to its association with coffee and cocoa, where it is used as a cover crop. Plantain is the leading food crop in forest areas [4].

In terms of food consumption, plantain ranks 4^th^ after rice, manioc, and cassava. It is consumed more in urban areas (53.4%) than in rural areas (46.6%). These fruits are most often used in the preparation of certain traditional dishes such as “foutou, aloco, claclo, and apiti” and other food products, including protein flour [5]. It is an excellent source of energy and nutrients (Mg, K, Ca, and P) that contribute significantly to food security in sub-Saharan Africa, where it is one of the main staple foods for over one hundred million people [6]. According to Thiémélé et al. [7], plantain is the 3^rd^ largest source of starch after yam and cassava. Despite relative self-sufficiency in plantain, the plantain sector faces a number of problems.

Unfortunately, 30% to 40% of production is lost before consumption. These losses are mainly due to poor harvesting, transport, and storage conditions [8]. According to Yao et al. [5], many efforts have been made by researchers to contribute to the conservation and processing of plantain, but much research remains to be done.

The aim of this study is to evaluate some microbiological and physicochemical parameters during fermentation for the production of an alcoholic beverage. It is part of the effort to reduce postharvest losses of plantain.

## 2. Materials and Methods

### 2.1. Sampling

Three plantain sellers in Korhogo's main market were randomly selected to collect plantain samples. Extremely ripe (black peel color) horn-type bananas were purchased from each seller and transported to the laboratory for the various experiments.

### 2.2. Production of Plantain Meal for Fermentation

In the laboratory, the plantains were first washed to remove the sand from the peel. They were then peeled and weighed. Nine hundred grams (900 g) of banana pulp were ground using an electric mixer (Binatone BLG-403, China) and distributed in 500 mL sterile bottles at a rate of 150 g of grind per bottle. Three batches of five (5) bottles of plantain must were prepared. Approximately 300 mL of water was then added to each bottle containing the banana crush. Once the water had been added, the mixture from one bottle of each batch was analyzed (T0). The other bottles of plantain must, sealed with aluminum foil and with a hole for the release of CO_2_, underwent fermentation for eight (8) days. Bottle content analyses were carried out at regular 2-day intervals. Three bottles are analyzed after two days of fermentation, i.e., one bottle from each batch.

### 2.3. Assessment of Physicochemical Parameters

#### 2.3.1. pH

pH was measured by immersing a calibrated pH meter electrode (pH-meter P604 consort, bio block, France) in 20 mL of plantain must in a beaker. The reading was repeated three times.

#### 2.3.2. Determination of Titratable Acidity

Acidity was determined by titration according to the method described by Amoa-Awua et al. [9]. To do this, 10 mL of each sample was titrated with a 0.1 N sodium hydroxide (NaOH) solution after adding 3 drops of 1% phenolphthalein. The operation was repeated three times, and the acidity level, expressed as a percentage, was determined according to the following formula:

(1) $$\begin{array}{c} \%\text{Titratable acidity}=\frac{V2\times N\times 0.09\times 100}{V1}, \end{array}$$

where *V*1 is the volume of solution dosed (mL), *V*2 is the volume of NaOH solution poured (mL), *N* is the normality of NaOH solution (meq./L), and 0.09 is the milliequivalent gram of lactic acid.

#### 2.3.3. Determination of Reducing Sugars

Reducing sugars were determined using the method described by Bernfeld [10]. A series of 1 : 10 dilutions were made for each sample in test tubes. To a volume of 0.5 mL of each dilution taken and introduced into the test tube, 1 mL of distilled water and 1 mL of 3,5-dinitrosalicylic acid (DNS) were added. The tubes are placed in a water bath for 5 minutes. After cooling, 7.5 mL of distilled water was added to each tube. The optical density of each tube was read at a wavelength of 540 nm using a spectrophotometer (GENESYS 5). A standard solution (glucose + fructose at 1 mg/mL) underwent the same protocol as the samples to obtain a calibration curve. Reducing sugar concentrations were determined from this calibration curve.

#### 2.3.4. Determination of Refractometric Dry Extract

The refractometric dry extract of plantain musts was determined using a refractometer. After cleaning the refractometer prism, a drop of plantain must was placed on it using a wash bottle. The prism containing the drop of must is closed with the lid of the instrument, and the refractometric dry extract value is read by looking through the eyepiece of the instrument.

#### 2.3.5. Alcohol Content

The alcohol content was determined using a manual refractometer with ATC. After cleaning the prism, a drop of plantain must is placed in it. The prism containing the drop of must was closed with the lid of the instrument, and the amount of alcohol was determined by looking through the eyepiece of the instrument.

### 2.4. Assessment of Microbiological Parameters

Mesophilic aerobic counts were carried out according to AFNOR NF V08-051 on plate count agar (Biokar, France). Inoculation was performed by incorporating 1 mL of inoculum from each dilution. Coliforms were counted in accordance with AFNOR standard, NF ISO 4832 July 1991; inoculation was carried out by incorporation onto Violet Red Bile Glucose Agar (Bio-Rad, France). Sulfite-reducing bacteria were counted by incorporation into test tubes containing Tryptone Sulfite Neomycin Agar (Bio-Rad, France). Streptococci were enumerated on Bile-Esculin-Azide Agar (Bio-Rad, France). Inoculation was carried out by incorporating 1 mL of inoculum from each dilution. Dichloran Rose Bengal Chloramphenicol (Biokar, France) was used for yeast counts. Inoculation was carried out by spreading according to standard NF V08-059.

The various microbial loads, expressed in cfu/mL, were calculated using the formula of ISO 7218.

(2) $$\begin{array}{c} N\left(\text{ufc}/\text{mL}\right)=\frac{\sum C}{\left(n1+0,1n2\right).d.V}\times 100. \end{array}$$

### 2.5. Statistical Analysis

The statistical processing of the results was carried out using Statistica software version 7.1. The software was used to calculate the standard deviation and the mean and to perform the normalized principal component analysis (NPCA).

## 3. Results

### 3.1. Physicochemical Parameters

#### 3.1.1. pH

**Figure 1:**
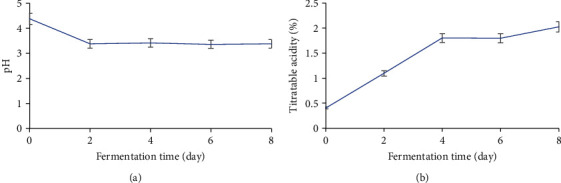
Variation in pH (a) and titratable acidity (b) during fermentation of plantain must.

Variations in pH and titratable acidity during fermentation are shown in Figure 1(a). The pH decreases during fermentation. The various values range from 4.37 to 3.36. The variation in pH is important between times T0 (4.37) and T2 (3.38) and remains practically constant between times T2 and T8.

#### 3.1.2. Titratable Acidity

Results for the variation in titratable acidity during fermentation are shown in Figure 1(b). Unlike pH, titratable acidity increases during fermentation. It rises from 0.4% (T0) to 2.02% (T8). A significant increase in acidity is observed between times T0 and T4. The variation is less significant between times T4 (1.8%) and T8 (2.02).

#### 3.1.3. Refractometric Dry Extract and Reducing Sugars

**Figure 2:**
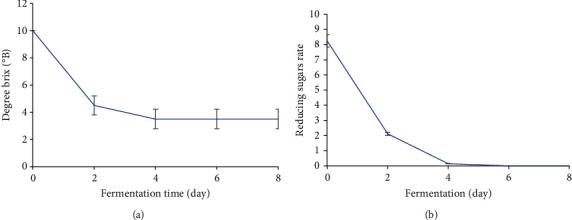
Variation in brix (a) and reducing sugars (b) during fermentation of plantain must.

Brix values and reducing sugar levels decrease during the fermentation of plantain must (Figure 2). Brix values fall sharply between day 0 and day 2 (10°B to 4.5°B), then stabilize between day 4 and day 8 at 3.5°B (Figure 2(a)). Reducing sugar levels drop from 8.25 to 0.15 mg/mL from day 0 to day 4 and become zero between days 6 and 8 (Figure 2(b)).

#### 3.1.4. Alcohol Content

**Figure 3:**
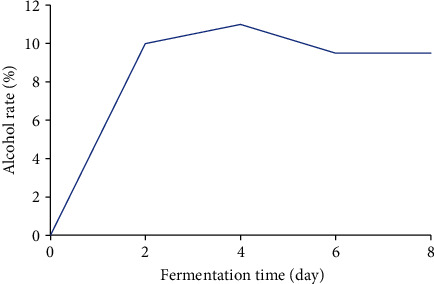
Variation in alcohol content during fermentation of plantain must.

Alcohol contents obtained during fermentation range from 0 to 11% (Figure 3). The highest alcohol content (11%) is obtained after four days of fermentation. After the fourth day, the alcohol content decreases and remains stable from the sixth to the eighth day.

### 3.2. Microbiological Parameters

#### 3.2.1. Aerobic Mesophilic Germs

**Figure 4:**
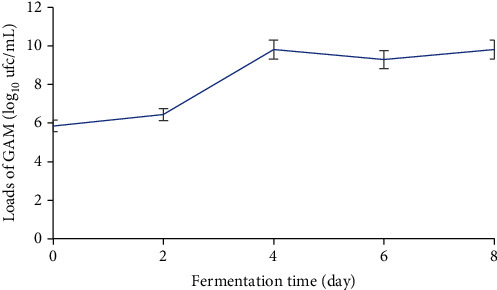
Variation in mesophilic aerobic germ loads during fermentation of plantain must.

During the fermentation of plantain must, aerobic mesophilic germ loads ranged from 4.84 to 9.8 log_10_ cfu/mL (Figure 4). The highest load was obtained after eight (8) days of fermentation. The variation in load was very marked between the first day (T0) and the fourth day of fermentation (T4). A slowdown in microbial growth was observed between the fourth (T4) and eighth (T8) days of fermentation.

#### 3.2.2. Total Coliforms and Sulfite-Reducing Bacteria

**Figure 5:**
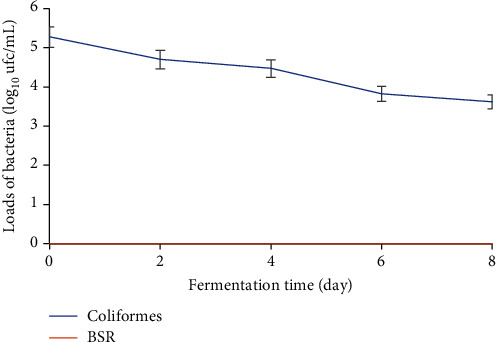
Variation in total coliform and sulfite-reducing bacteria loads during fermentation of plantain must.

The evolution of total coliform and sulfite-reducing bacteria loads during fermentation is shown in Figure 5. Total coliform loads decrease during fermentation. They drop from 5.27 log_10_ cfu/mL (day one) to 3.61 log_10_ cfu/mL (day eight). A significant drop in these loads is observed between T0 (5.27 log_10_ cfu/mL) and T2 (4.7 log_10_ cfu/mL) before slowing down between T2 and T4 (4.47 log_10_ cfu/mL). A significant drop in loads was also observed between T4 and T8 (3.61 log_10_ cfu/mL). Sulfite-reducing bacteria were absent during fermentation.

#### 3.2.3. Yeasts

**Figure 6:**
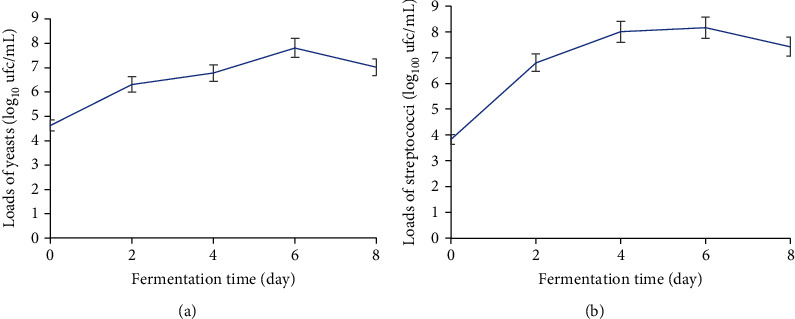
Variation in yeast (a) and streptococci (b) loads during fermentation of plantain must.

Yeast loads varied from 4.63 to 7.81 log_10_ cfu/mL during fermentation (Figure 6(a)). A progressive increase in yeast loads was observed between time T0 (4.63 log_10_ cfu/mL) and time T6 (7.81 log_10_ cfu/mL). Growth is most pronounced between T0 and T2. Growth peaks (7.81 log_10_ cfu/mL) on the sixth day of fermentation before dropping slightly (7.01 log_10_ cfu/mL) on the eighth day.

#### 3.2.4. Streptococci

Streptococcal loads ranged from 3.82 to 8.15 log_10_ cfu/mL during fermentation (Figure 6(b)). The variation in streptococcal load is identical to that of yeast. Indeed, a progressive growth of streptococci is observed between times T0 and T6, where it reaches its peak (8.15 log_10_ cfu/mL) before decreasing to 7.42 and 8.15 log_10_ cfu/mL on day eight.

### 3.3. Statistical Analysis

**Figure 7:**
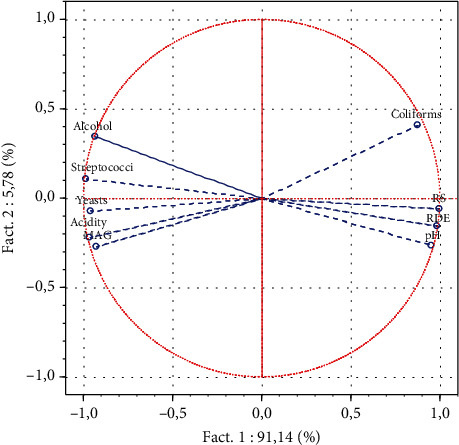
Correlation circle between physicochemical and microbiological parameters.

A normalized principal component analysis (NPCA) was carried out to highlight the relationships between the various parameters studied. The factorial plan summarizes 96.92% of the overall information, i.e., 91.14% for the *F*1 axis and 5.78% for the *F*2 axis (Figure 7). On the *F*1 axis, two opposite groups of parameters stand out, showing a negative correlation between them. Group 1 is made up of parameters (coliforms, reducing sugars (RS), refractometric dry extract (RDE), and pH) and group 2 (alcohol, streptococci, yeasts, acidity, and mesophilic aerobic germs (MAG)).

## 4. Discussion

Physicochemical analyses showed a progressive acidification of plantain must during fermentation. This result corroborates that of Tsiba-Nkoli [11] in the study carried out during the production of wine from plantain. The pH of the banana must obtained before fermentation is identical to that of Ourega et al. [12]. According to these authors, the acid pH of banana pulps reflects the presence of organic acid in the pulp at a very advanced stage of ripening. Unlike pH, titratable acidity increases during fermentation. Variation in pH is strongly linked to acid production during fermentation, hence the strong negative correlation between these two parameters. The acidification of musts during fermentation could be linked to the action of lactic acid bacteria. According to Benyoucef [13], lactic acid bacteria have the ability to produce organic acid from carbon through the fermentation process. The strong correlation between streptococci and titratable acidity testifies to the involvement of lactic bacteria in the acidification of banana must. According to Dahouenon-Ahoussi et al. [14], an acidic environment is favorable to the development of yeasts that ensure alcoholic fermentation. Thus, like titratable acidity, an increase in alcohol content was recorded, strongly negatively correlated with refractometric dry extract and reducing sugar levels. According to Aka et al. [15], yeasts use fermentable sugars, which they convert into alcohol and carbon dioxide. Sugars are essential elements in fermentation, as they are the main source of energy for yeast metabolism. In addition to yeast, lactic acid bacteria also transform sugars into organic acids. The evolution of sugar levels is comparable to that of Gbohaida et al. [16] for the study on the fermentative power of *Saccharomyces cerevisiae* and *S. carlsbergensis* in the production of bioethanol from cashew apple juice. The streptococci in this study could belong to the heterofermentative lactic acid bacteria group, given the strong correlation between these bacteria and alcohol content. The alcohol levels obtained in this study are higher than those obtained by Ourega et al. [12], who obtained alcohol levels of between 3.6% and 6.77% in beers produced from plantain waste. On the other hand, the results obtained are lower than those of Habamubgu et al. [17], who obtained results of between 20% and 25% in the study of local beverages newly produced by the population of South Kivu. Alcohol levels peak after four (4) days of fermentation, drop between days four and six, and remain constant between days six and eight. This drop in alcohol content could be linked to the transformation of alcohol produced during fermentation into acetic acid by acetic bacteria. Unlike mesophilic aerobic germs, yeasts, and streptococci, total coliform loads decrease during plantain must fermentation. According to Karamoko [18], lactic acid bacteria produce acids that allow acidification of the medium, and this could be the cause of the decrease in coliform loads. Lactic and acetic acids play an important role in inhibiting the growth of microorganisms [19]. The increase in mesophilic aerobic germ loads during fermentation is linked to the composition of the microorganisms in this group. Indeed, the group of aerobic mesophilic germs also includes fermentative microorganisms.

## 5. Conclusion

The aim of this study was to evaluate physicochemical and microbiological parameters during spontaneous fermentation of plantain must. The highest alcohol levels are obtained after four (4) days of fermentation. The evolution of streptococci during fermentation is identical to that of yeasts. Both microorganisms reach maximum growth after six (6) days of fermentation of plantain must. A strong positive correlation is observed between fermentation germs (yeasts, streptococci) and the production of alcohol and acid. Both yeasts and streptococci are involved in plantain must fermentation.

## Data Availability

The data used to support the findings of this study are included in the article.
